# Sarcopenia is associated with the Geriatric Nutritional Risk Index in elderly patients with poorly controlled type 2 diabetes mellitus

**DOI:** 10.1111/jdi.13792

**Published:** 2022-03-24

**Authors:** Shun Matsuura, Koji Shibazaki, Reiko Uchida, Yukiko Imai, Takuya Mukoyama, Shoko Shibata, Hiroshi Morita

**Affiliations:** ^1^ 37070 Division of Diabetes Endocrinology Medicine Fujieda Municipal General Hospital Fujieda Japan; ^2^ 37070 Division of Respiratory Internal Medicine Fujieda Municipal General Hospital Fujieda Japan

**Keywords:** Geriatric nutritional risk index, Sarcopenia, Type 2 diabetes mellitus

## Abstract

**Aims/Introduction:**

Diabetes and sarcopenia have a two‐way relationship with each other with advanced age. Additionally, malnutrition is correlated with a higher risk of sarcopenia in elderly patients. This study evaluated the association between sarcopenia and geriatric nutritional risk index (GNRI) in elderly patients with type 2 diabetes mellitus.

**Materials and Methods:**

Patients with type 2 diabetes mellitus aged ≥60 years were recruited from June 2018 to August 2020. This study analyzed 234 patients, who completed a physical performance test required for the diagnosis of sarcopenia. To investigate the effect of GNRI on sarcopenia, logistic regression analyses was used.

**Results:**

Patients with sarcopenia were significantly older with a lower body mass index (BMI) and GNRI compared with normal patients. The GNRI showed a positive correlation with the skeletal muscle index (SMI) and handgrip strength (SMI: *R* = 0.486, *P* < 0.001 for male; *R* = 0.589, *P* < 0.001 for female, handgrip strength: *R* = 0.470, *P* < 0.001 for male, *R* = 0.364, *P* < 0.001 for female). In the multivariate logistic regression model, a higher GNRI was associated with a lower risk of sarcopenia in older men and women with diabetes (adjusted odds ratio [OR], 0.892; 95% confidence interval [CI], 0.839–0.948 for male; adjusted OR, 0.928; 95% CI, 0.876–0.982 for female). One year of diabetes treatment improved the GNRI in the sarcopenia group with type 2 diabetes mellitus.

**Conclusions:**

A low GNRI was associated with an increased risk of sarcopenia in elderly patients with type 2 diabetes mellitus. Treatment with glucose‐lowering drugs improved the GNRI in the sarcopenia group.

## Graphical abstract

Low GNRI levels were associated with an increased risk of sarcopenia in elderly patients with type 2 diabetes mellitus. Treatment with glucose‐lowering drugs improved the GNRI in the male sarcopenia group.

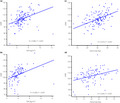

## INTRODUCTION

Sarcopenia is a condition characterized by significant decreases in skeletal muscle mass and function with age. Sarcopenia is always considered to be multifactorial and associated with multiple chronic diseases. Sarcopenia accelerates the progress of metabolic diseases. Patients with diabetes lose muscle mass, muscular strength, and physical ability decreases, resulting in sarcopenia^1^, ^2^, ^3^. A previous study reported that the prevalence of sarcopenia is significantly higher in patients with type 2 diabetes mellitus than in the general group^4^, ^5^. Type 2 diabetes mellitus and sarcopenia are widespread conditions at advanced age with a bidirectional relationship^6^.

A few studies have referred to the relation between malnutrition and the increased risk of sarcopenia in elderly adults^7^, ^8^. The Geriatric Nutritional Risk Index (GNRI), which is calculated using serum albumin levels and body mass index (BMI)^9^, is an objective and simple screening tool that has garnered considerable attention as a significant predictor of prognosis for patients with chronic disease^10^, ^11^, ^12^. The GNRI is commonly used to evaluate elderly patients. Malnutrition is frequently found in elderly individuals, and the prevention of malnutrition is an important treatment for sarcopenic elderly patients with type 2 diabetes mellitus. However, it is not clear whether sarcopenia is associated with the nutritional status of elderly patients with a diabetic condition.

Our goal in this study was to assess the association between sarcopenia and GNRI in elderly patients with type 2 diabetes.

## PATIENTS AND METHODS

### Study population

This retrospective analysis used data from elderly (≥60 years old) outpatients with type 2 diabetes mellitus visiting the Fujieda Municipal General Hospital (Shizuoka, Japan) from February 2018 to August 2020. This study analyzed 234 patients who finished a physical performance test required for the diagnosis of sarcopenia. Patients who had type 1 diabetes mellitus, chronic pancreatic disease, cirrhosis, dialysis, or known advanced cancer were excluded. During the first year after administration, attending physicians treated the patients according to the standards of medical care in type 2 diabetes, including a proper diet, educational admission, and medications. The study was approved by the medical ethics committee of the Fujieda Municipal General Hospital (R03‐17).

### Clinical data collection

The electronic medical record system of the patients was screened to assess diabetes‐related factors. These factors include glycated hemoglobin (HbA1c), body mass index (BMI), disease duration, degree of diabetic retinopathy progression, and medication history. In addition, routine blood tests were done, such as serum albumin level, high‐density lipoprotein cholesterol (HDL), low‐density lipoprotein cholesterol (LDL), and estimated glomerular filtration rate.

### Assessment of sarcopenia

As an indicator of physical ability, two variables were used: the limb skeletal muscle mass and the handgrip strength. The skeletal muscle mass index was measured using a bioelectrical impedance analysis (InBody270; InBody Japan Inc, Tokyo, Japan). The skeletal muscle mass index (SMI) was measured by dividing the limb skeletal muscle mass (kg) by the square of the height (m^2^). If we had males with SMI <7.0 kg/m^2^ or females with <5.7 kg/m^2^, we considered them to have a low muscle mass. In contrast, we assessed the grip strength using a handgrip dynamometer (TKK5001; Takei Scientific Instruments, Tokyo, Japan). As a result, a handgrip strength of <28 kg for males and <18 kg for females were considered as signs of low muscle strength. The diagnosis of sarcopenia was confirmed according to the updated consensus on sarcopenia diagnosis and treatment issued by the Asian Working Group for Sarcopenia in 2019^8^.

### Nutritional assessment using GNRI

To calculate GNRI, the equation: 14.89 × serum albumin (g/dL) + 41.7 × (body weight/ideal body weight) was used. The ideal body weight was calculated using height and BMI (22.0 kg/m^2^). However, we set the body weight/ideal body weight at 1 if the patient’s body weight exceeded their ideal body weight. The aim and strategy behind this step were defined in a previous study^9^.

### Statistical analysis

To present our results, we used mean ± standard deviation. However, to compare two or three groups, Fisher’s exact test for discrete variables was used and one‐way analysis of variance (ANOVA) for continuous variables. Using Wilcoxon’s signed‐rank test, the significance of differences in the continuous variables was assessed. Factor analysis was done to assess the factors related to sarcopenia using multivariate logistic regression analysis. However, this was done using potential factors according to the *P* < 0.20 results of the univariate logistic regression analysis. The results of the regression modeling are presented as the odds ratio (OR) and 95% confidence interval (CI). All statistical tests were two‐way. Statistical analyses were performed using EZR (Saitama Medical Center, Jichi Medical University)^13^, which is a graphical user interface for R (R Foundation for Statistical Computing, ver. 3.4.1).

## RESULTS

### Patient characteristics

This study evaluated the baseline characteristics in 234 patients aged ≥60 years and over (Table 1). The overall prevalence of sarcopenia was 24.7%, with 22.3% for males and 28.4% for females. The mean age of the sarcopenia group was significantly higher than that of the normal group (*P* < 0.01). Patients with sarcopenia had a lower BMI and GNRI than those without sarcopenia. No difference was noted in the metabolic control or frequency of diabetes complications between the sarcopenia and normal groups. Moreover, 55.1% of the patients (129 patients) were being treated with glucose‐lowering drugs – 13.2% were treated with insulin, 22.6% with biguanides, and 32.1% with DPP‐4 inhibitors. There was no statistically significant difference in the therapeutic agents between the groups.

**Table 1:** The clinical characteristics of the patients

Factor	All patients			Male			Female		
	Normal	Sarcopenia	*P*‐value	Normal	Sarcopenia	*P*‐value	Normal	Sarcopenia	*P*‐value
	*n* = 176	*n* = 58		*n* = 108	*n* = 31		*n* = 68	*n* = 27	
Age (years)	70.3 ± 6.4	75.71 ± 7.54	<0.001	70.8 ± 6.2	75.5 ± 7.4	0.001	69.3 ± 6.9	75.9 ± 7.7	<0.001
Diabetes duration	7.1 ± 8.9	9.8 ± 10.0	0.048	7.9 ± 9.7	10.6 ± 10.5	0.196	5.6 ± 7.1	8.9 ± 9.5	0.073
Dyslipidemia (%)	91 (51.7)	24 (41.4)	0.178	49 (45.4)	9 (29.0)	0.148	41 (60.3)	15 (55.6)	0.818
Hypertension (%)	112(63.6)	29 (50.0)	0.088	67 (62.0)	16 (51.6)	0.307	45 (66.2)	13 (48.1)	0.161
Nephropathy (%)									
Stage 1	124 (70.5)	37 (63.8)	0.345	77 (71.3)	17 (54.8)	0.152	46 (67.6)	20 (74.1)	0.229
Stage 2	38 (21.6)	12 (20.7)		19 (17.6)	8 (25.8)		19 (27.9)	4 (14.8)	
Stage 3	10 (5.7)	7 (12.1)		9 (8.3)	6 (19.4)		2 (2.9)	1 (3.7)	
Stage 4	4 (2.3)	2 (3.4)		3 (2.8)	0 (0.0)		1 (1.5)	2 (7.4)	
Retinopathy (%)									
Non	135 (76.7)	46 (79.3)	0.722	85 (78.7)	23 (74.2)	0.182	51 (75.0)	23 (85.2)	0.497
Simple	29 (16.5)	10 (17.2)		16 (14.8)	8 (25.8)		12 (17.6)	2 (7.4)	
Proliferative	12 (6.8)	2 (3.4)		7 (6.5)	0 (0.0)		5 (7.4)	2 (7.4)	
HbA1c (%)	10.0 ± 2.4	10.3 ± 2.5	0.317	10.1 ± 2.6	11.0 ± 2.9	0.124	9.7 ± 2.0	9.6 ± 1.8	0.833
eGFR (mL/min/1.73 m^2^)	70.1 ± 23.9	73.3 ± 28.1	0.392	71.2 ± 24.2	72.6 ± 26.3	0.779	67.9 ± 23.4	74.1 ± 30.7	0.29
HDL (mg/dL)	56.9 ± 16.7	61.5 ± 19.3	0.089	55.9 ± 15.9	59.1 ± 16.7	0.338	58.7 ± 18.0	64.2 ± 21.9	0.21
LDL (mg/dL)	124.2 ± 40.6	117.8 ± 46.3	0.321	116.5 ± 34.2	114.0 ± 29.2	0.712	136.8 ± 46.1	122.2 ± 60.6	0.208
Albumin (g/dL)	4.0 ± 0.4	3.8 ± 0.5	0.003	4.1 ± 0.4	3.8 ± 0.5	0.005	4.0 ± 0.4	3.9 ± 0.5	0.165
BMI (kg/m^2^)	23.9 ± 4.4	20.5 ± 3.1	<0.001	23.3 ± 4.0	20.1 ± 3.1	<0.001	24.8 ± 4.8	20.9 ± 3.0	<0.001
Handgrip strength (kg)	29.4 ± 7.6	18.9 ± 5.5	<0.001	33.3 ± 5.9	22.2 ± 4.8	<0.001	22.7 ± 5.3	14.8 ± 2.9	<0.001
SMI (kg/m^2^)	6.8 ± 1.2	5.6 ± 0.7	<0.001	7.2 ± 1.0	6.0 ± 0.6	<0.001	6.2 ± 1.2	5.2 ± 0.4	<0.001
GNRI	106.4 ± 9.9	96.7 ± 10.9	<0.001	105.6 ± 9.3	95.5 ± 11.0	<0.001	107.8 ± 10.2	98.1 ± 10.9	<0.001
Insulin (%)	23 (13.1)	8 (13.8)	1.000	14 (13.0)	5 (16.1)	0.767	10 (14.7)	3 (11.1)	0.752
Biguanide (%)	39 (22.2)	14 (24.1)	0.857	27 (25.0)	6 (19.4)	0.635	12 (17.6)	8 (29.6)	0.264
DPP4 inhibitor (%)	52 (29.5)	23 (39.7)	0.194	36 (33.3)	14 (45.2)	0.289	16 (23.5)	9 (33.3)	0.439
Sulfonylurea (%)	41 (23.3)	16 (27.6)	0.597	25 (23.1)	9 (29.0)	0.487	16 (23.5)	7 (25.9)	0.796
SGLT2 inhibitor (%)	16 (9.1)	3 (5.2)	0.419	8 (7.4)	3 (9.7)	0.709	8 (11.8)	0 (0.0)	0.100
Alfa‐GI (%)	14 (8.0)	9 (15.5)	0.125	10 (9.3)	5 (16.1)	0.325	4 (5.9)	4 (14.8)	0.218
Glinide (%)	6 (3.4)	6 (10.3)	0.078	5 (4.6)	4 (12.9)	0.112	1 (1.5)	2 (7.4)	0.194
GLP1 RA (%)	7 (4.0)	0 (0.0)	0.198	2 (1.9)	0 (0.0)	1.000	5 (7.4)	0 (0.0)	0.317
TZD (%)	5 (2.8)	3 (5.2)	0.413	2 (1.9)	2 (6.5)	0.215	3 (4.4)	1 (3.7)	1.000
No treatment (%)	80 (45.5)	25 (43.1)	0.764	47 (43.5)	12 (38.7)	0.684	33 (48.5)	13 (48.1)	1.000

BMI, body mass index; DPP4, dipeptidyl peptidase 4; eGFR, estimated glomerular filtration rate; GI, glucosidase inhibitor; GLP‐1RA, glucagon‐like peptide‐1 receptor agonists; GNRI, geriatric nutritional risk index; HbA1c, glycated hemoglobin; HDL, high‐density lipoprotein cholesterol; LDL, low density lipoprotein cholesterol; SGLT2, sodium–glucose cotransporter 2; SMI, smooth muscle index; TZD, Thiazolidinedione.

### Correlations between GNRI and the components of sarcopenia

**Figure 1:**
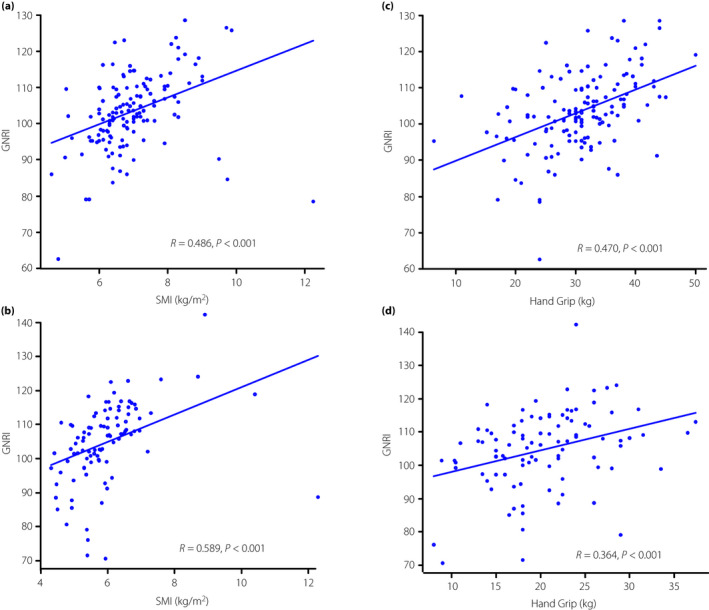
(a) Male and (b) female, correlation between GNRI and SMI. (c) Male and (d) female, correlation between GNRI and handgrip strength. SMI, skeletal muscle index; GNRI, geriatric nutritional risk index.

The GNRI showed a positive correlation with SMI and the handgrip strength for males (SMI: *R* = 0.486, *P* < 0.001; handgrip: *R* = 0.470, *P* < 0.001; Figure 1a,c). The GNRI showed a weak positive correlation with SMI and handgrip strength for females (SMI: *R* = 0.589, *P* < 0.001; *R* = 0.364, *P* < 0.001; Figure 1b,d).

### Analysis results of multivariate logistic regression models for the risk factors of sarcopenia

The results of multivariate logistic regression model are shown in Table 2. In univariate analysis, age, diabetes duration, dyslipidemia, nephropathy, GNRI, and glinide use were significant factors for males, and age, diabetes duration, serum levels of LDL, GNRI, glinide, and glucosidase inhibitor use were significant factors for females. It was found that older age (OR: 1.110, 95% CI: 1.030–1.190 for males; OR: 1.100, 95% CI: 1.010–1.190 for females) was significantly correlated with a high risk of sarcopenia. Higher GNRI appeared to prevent sarcopenia in elderly males and females with type 2 diabetes mellitus (OR: 0.892, 95% CI: 0.839–0.948 for males; OR: 0.928, 95% CI: 0.876–0.982 for females). However, the other indicators of factors were not associated with sarcopenia with type 2 diabetes mellitus.

**Table 2:** Logistic regression models for risk factors associated with sarcopenia

	Odds ratio	95%CI	*P*‐value
Male			
Age (years)	1.110	1.030–1.190	0.007
Diabetes duration (years)	1.020	0.974–1.070	0.401
Dyslipidemia	0.902	0.304–2.670	0.852
Nephropathy	1.190	0.646–2.190	0.578
GNRI	0.892	0.839–0.948	<0.001
Glinide	2.210	0.437–11.20	0.337
Female			
Age (years)	1.110	1.010–1.190	0.024
Diabetes duration (years)	1.040	0.965–1.120	0.323
Hypertension	0.386	0.122–1.220	0.105
LDL (mg/dL)	0.999	0.987–1.010	0.900
GNRI	0.928	0.876–0.982	0.009
Alfa‐GI	1.570	0.082–29.60	0.765

GI, glucosidase inhibitor; GNRI, geriatric nutritional risk index; LDL, low density lipoprotein cholesterol.

### Glucose‐lowering drugs and sarcopenia in patients with type 2 diabetes mellitus

The longitudinal changes in HbA1c and GNRI were studied. Table 3 shows the newly added drugs during the first year, and drugs used at the time of the first referral were excluded. For additional treatments, glinide was often added in the male sarcopenia group. Table 4 shows the subgroup analyses comparing the HbA1c value and GNRI assessed by sarcopenia. The mean HbA1c score in all groups decreased significantly after the administration of glucose‐lowering drugs. The changes in GNRI after the administration of the glucose‐lowering drugs were increased significantly in the sarcopenia group (from 96.7 ± 10.9 to 99.7 ± 8.2, *P* = 0.009), whereas the changes in GNRI were comparable in the normal group.

**Table 3:** Additional glucose‐lowering drugs at administration

	All patients (*n* = 234)			Male (*n* = 139)			Female (*n* = 95)		
	Normal	Sarcopenia	*P*‐value	Normal	Sarcopenia	*P*‐value	Normal	Sarcopenia	*P*‐value
Insulin (%)	22 (14.5)	11 (22.0)	0.269	17 (18.1)	8 (30.8)	0.188	5 (8.6)	3 (12.5)	0.687
Biguanide (%)	32 (23.4)	12 (27.3)	0.687	15 (18.5)	7 (28.0)	0.397	17 (29.3)	5 (26.3)	1.000
DPP‐4 inhibitor (%)	88 (69.8)	25 (71.4)	1.000	49 (67.1)	10 (58.8)	0.576	39 (73.6)	15 (83.3)	0.531
SGLT2 inhibitor (%)	14 (8.8)	4 (7.3)	1.000	8 (8.0)	4 (14.3)	0.294	6 (9.8)	0 (0.0)	0.171
Alfa‐GI (%)	3 (1.8)	3 (6.0)	0.143	2 (2.0)	3 (11.1)	0.066	1 (1.5)	0 (0.0)	1.000
Glinide (%)	12 (7.1)	8 (15.4)	0.094	6 (5.8)	6 (22.2)	0.018	6 (9.1)	2 (8.0)	1.000
GLP1 RA (%)	13 (7.7)	7 (12.1)	0.420	10 (9.4)	5 (16.1)	0.329	3 (6.2)	2 (7.4)	0.637

DPP4, dipeptidyl peptidase 4; GI, glucosidase inhibitor; GLP‐1RA, glucagon‐like peptide‐1 receptor agonists; SGLT2, sodium–glucose cotransporter 2.

**Table 4:** Changes in HbA1c and GNRI to the effect of additional glucose‐lowering drug treatment

	HbA1c (%)			GNRI		
	Baseline	12 months	*P*‐value	Baseline	12 months	*P‐*value
All patients						
Normal	10.0 ± 2.4	7.3 ± 1.1	<0.001	106.6 ± 9.9	106.8 ± 9.8	0.699
Sarcopenia	10.3 ± 2.5	7.5 ± 1.1	<0.001	96.7 ± 10.9	99.7 ± 8.2	0.007
Male						
Normal	10.1 ± 2.6	7.2 ± 1.1	<0.001	105.6 ± 9.3	106.2 ± 9.3	0.349
Sarcopenia	11.0 ± 2.9	7.6 ± 1.3	<0.001	95.5 ± 11.0	99.1 ± 8.1	0.019
Female						
Normal	9.7 ± 2.0	7.3 ± 1.0	<0.001	107.8 ± 10.2	107.8 ± 10.5	0.888
Sarcopenia	9.6 ± 1.8	7.3 ± 0.8	<0.001	98.1 ± 10.9	100.6 ± 8.3	0.069

GNRI, geriatric nutritional risk index; HbA1c, glycated hemoglobin.

## DISCUSSION

This study found that a low GNRI score in elderly patients with type 2 diabetes mellitus was a risk factor for sarcopenia. In addition, one year of diabetes treatment improved GNRI in the sarcopenia group with type 2 diabetes mellitus. To the best of our knowledge, the association between GNRI and sarcopenia in adults with type 2 diabetes mellitus has not yet been examined.

A previous study reported a correlation between malnutrition and decreased muscle strength in elderly subjects^14^. Multiple factors can contribute to the development of sarcopenia, including aging, inactivity, malnutrition, and chronic disease^15^. However, malnutrition is also one of them^16^, ^17^. Additionally, our result indicated the same relation between malnutrition due to low GNRI and sarcopenia. A high prevalence of sarcopenia has been observed in individuals with type 2 diabetes mellitus with poor nutritional status^18^. Therefore, proper nutritional management plays an important role in reducing the risk of sarcopenia in elderly patients with type 2 diabetes mellitus.

Evaluations by the Global Leadership Initiative on Malnutrition criteria, the European Society of Clinical Nutrition and Metabolism criteria, and a mini nutrition assessment have been reported as nutritional indicators of sarcopenia^17^, ^18^. A previous study showed that a higher BMI and skeletal muscle index decrease the probability of developing sarcopenia^19^. Moreover, BMI is significantly lower in type 2 diabetes mellitus individuals with sarcopenia than in those without it^20^. The Japan Diabetes Society has set a target BMI of 22–25 for elderly patients. The rationale is that the target body weight is calculated by considering that BMI values associated with mortality from all causes changes with age^21^. To assess the nutritional status, serum albumin is considered the simplest and most valuable tool^22^. On the other hand, hypoalbuminemia is strongly correlated with complications and mortality in the elderly^23^, ^24^, ^25^. Markers of chronic subclinical inflammation, such as low levels of serum albumin, were associated with an increased risk of type 2 diabetes^26^. A low serum albumin and chronic inflammation in type 2 diabetes mellitus caused muscle weakness and atrophy^27^, ^28^. The GNRI is considered multidimensional because it reflects both an anthropometric factor (BMI) and a serum marker (albumin). The GNRI score showed a good ability to identify elderly patients who are sarcopenic. In this study, GNRI was correlated with SMI and handgrip strength. Previous studies have highlighted the significance of GNRI in nutrition‐related risk assessments of elderly people and its close relationship with muscle function^29^, ^30^. There are some indications that a low GNRI with type 2 diabetes mellitus could be associated with worse osteoporosis and foot disease^31^, ^32^. Diabetic complications can impair the patient’s quality of life and increase morbidity. We previously reported that patients with advanced lung cancer with low GNRI scores were significantly associated with a relatively poor performance status^33^. Low GNRI may serve as one explanation for why malnutrition is associated with poor performance status. The prevention of malnutrition in elderly patients with type 2 diabetes might prevent sarcopenia and lead to good physical performance.

A high glycemic level in elderly patients with diabetes mellitus was associated with low muscle mass and muscle quality^34^, ^35^. Likewise, poor glycemic control in patients with type 2 diabetes was reported to be a risk factor for sarcopenia^36^. Glucose‐lowering drugs for type 2 diabetes mellitus can have a beneficial effect on some factors possibly involved in sarcopenia^37^. Our study showed that the effects of diabetes treatment after 12 months improved the nutritional status in elderly sarcopenic diabetic patients. Recently, a study reported that glucose‐lowering interventions are effective against skeletal muscle mass and sarcopenia^38^. Reports indicate that inflammatory markers, such as low serum albumin, are a risk for diabetes^39^. In our study, the improvement of GNRI was attributed to improving glycemic control in patients with sarcopenia and diabetes, increased body weight, including skeletal muscle mass, and improving low‐grade inflammation increased the serum albumin levels (Table S2). Nutritional status may be improved with a good management approach to sarcopenia and diabetes. As a result, that might participate in maintaining muscular function and increasing the serum albumin level^40^. Sarcopenia in elderly patients with type 2 diabetes mellitus might require adequate glycemic control with hypoglycemic agents rather than an overly restricted dietary pattern.

This study has some limitations. First, our study was limited by its retrospective study design, and the sample size was modest. The study was conducted in a single institution, and determining general relationships was not always possible. Second, we did not have data on the patients’ walking speed, which might be involved in sarcopenia with type 2 diabetes mellitus. Third, our cohort of patients with poorly controlled type 2 diabetes may give different results from the general cohort of elderly type 2 diabetes patients. Because approximately half of the diabetic patients were untreated, it is considered that the therapeutic effect of the glucose‐lowering drugs was remarkable. If the patients with type 2 diabetes had been better controlled, the association between sarcopenia and GNRI may also have been different. Fourth, glinide was frequently selected for the male sarcopenia group. The reason may be that glinide was selected because postprandial hyperglycemia is elevated in sarcopenic patients. We further examined changes in GNRI for each of the added drugs (Table S1); however, no significant difference was noted between the drugs. The small number of cases limits the interpretation of the subgroup analysis. Fifth, nutritional and inflammatory assessments with metrics other than the GNRI were not performed due to the limited information included in this study. Moreover, we did not consider physical exercise and food intake. Sixth, sarcopenia with reduced skeletal muscle mass is thought to be associated with the total body weight. GNRI is a weight‐based measurement, and the association between GNRI and sarcopenia in this study may be due to body weight aspects. Further prospective trials are now warranted to examine the beneficial effects of an increase in GNRI for preventing sarcopenia in patients with type 2 diabetes mellitus.

In conclusion, our study is the first to assess sarcopenia risk and GNRI. However, GNRI correlates positively with sarcopenia in patients with type 2 diabetes mellitus. Additionally, the administration of the glucose‐lowering drugs improves GNRI in sarcopenic patients and might decrease the risk of sarcopenia.

## DISCLOSURE

The authors declare no conflict of interest.

Approval of the research protocol: N/A.

Informed consent: This study was approved by the Institutional Review Board of Fujieda Municipal General Hospital (R03‐17). The Institutional Review Board waived the requirement for informed consent from patients because of the retrospective design of the research.

Approval date of registry and the registration no. of the study/trial: N/A.

Animal studies: N/A.

## Supporting information


**Table S1** | Changes in HbA1c and GNRI on the effect of subgroup of additional glucose‐lowering drug treatment
**Table S2** | Changes in albumin and BMI on the effect of additional glucose‐lowering drugs treatment
