# Supplementary material for: Sarcopenia is associated with the Geriatric Nutritional Risk Index in elderly patients with poorly controlled type 2 diabetes mellitus
Source: J Diabetes Investig. 2022 Mar 24;13(8):1366–73. doi: 10.1111/jdi.13792 (PMC9340875; doi:10.1111/jdi.13792)
Supplement: Supplementary file 1 — Table S1 | Changes in HbA1c and GNRI on the effect of subgroup of additional glucose‐lowering drug treatment Table S2 | Changes in albumin and BMI on the effect of additional glucose‐lowering drugs treatment [file JDI-13-1366-s001.docx]

Supplementary Table 1. Changes in HbA1c and GNRI to the effect of subgroup of additional glucose-lowering drugs treatment

|  | **HbA1c** | | |  | **GNRI** | | |
| --- | --- | --- | --- | --- | --- | --- | --- |
|  | **baseline** | **12 months** | ***p*-value** |  | **baseline** | **12 months** | ***p-*value** |
| **Male** |  |  |  |  |  |  |  |
| **Normal** |  |  |  |  |  |  |  |
| **Insulin** | **11.7 ± 3.5** | **7.1 ± 1.0** | **<0.001** |  | **104.4 ± 10.1** | **104.0 ± 8.7** | **0.487** |
| **Biguanide** | **13.2 ± 3.3** | **6.91± 0.7** | **<0.001** |  | **102.0 ± 8.4** | **103.8 ± 7.6** | **0.135** |
| **DPP-4 inhibitor** | **11.0 ± 3.0** | **6.88 ± 0.9** | **<0.001** |  | **104.7 ± 9.2** | **106.4 ± 9.5** | **0.148** |
| **SGLT2 inhibitor** | **9.4 ± 1.6** | **7.8 ± 0.6** | **0.107** |  | **107.1 ± 4.7** | **108.3 ± 5.0** | **0.383** |
| **Glinide** | **8.9 ± 0.7** | **7.30 ± 1.4** | **0.092** |  | **99.1 ± 8.3** | **97.0 ± 3.9** | **0.563** |
| **GLP1 RA** | **9.6 ± 1.8** | **8.15 ± 1.3** | **0.125** |  | **106.1 ± 10.3** | **107.6 ± 8.9** | **0.275** |
| **Sarcopenia** |  |  |  |  |  |  |  |
| **Insulin** | **11.9 ± 3.7** | **7.5 ± 0.9** | **0.007** |  | **90.4 ± 13.4** | **96.1 ± 9.1** | **0.461** |
| **Biguanide** | **12.4 ± 3.1** | **7.5 ± 1.4** | **0.015** |  | **96.1 ± 8.1** | **101.0 ± 8.1** | **0.078** |
| **DPP-4 inhibitor** | **10.9 ± 2.6** | **7.3 ± 1.1** | **0.008** |  | **97.0 ± 9.9** | **99.6 ± 8.0** | **0.091** |
| **SGLT2 inhibitor** | **11.3 ± 2.5** | **8.9 ± 2.0** | **0.625** |  | **109.4 ± 8.9** | **110.7 ± 7.8** | **0.625** |
| **Glinide** | **12.2 ± 3.1** | **7.6 ± 1.3** | **0.031** |  | **92.8 ± 15.8** | **98.9 ± 8.9** | **0.156** |
| **GLP1 RA** | **10.9 ± 0.9** | **7.1 ± 0.4** | **0.058** |  | **97.7 ± 8.2** | **99.3 ± 4.7** | **1.000** |
| **Female** |  |  |  |  |  |  |  |
| **Normal** |  |  |  |  |  |  |  |
| **Insulin** | **11.3 ± 1.8** | **7.9 ± 0.7** | **0.062** |  | **98.3 ± 19.9** | **100.2 ± 15.1** | **0.812** |
| **Biguanide** | **11.1 ± 2.2** | **7.4 ± 1.1** | **<0.001** |  | **10.6.1 ± 5.1** | **107.2 ± 6.0** | **0.190** |
| **DPP-4 inhibitor** | **10.3 ± 2.0** | **7.0 ± 0.9** | **<0.001** |  | **106.2 ± 8.1** | **106.7 ± 10.2** | **0.296** |
| **SGLT2 inhibitor** | **9.6 ± 1.4** | **7.3 ± 0.4** | **0.031** |  | **115.1 ± 15.3** | **114.5 ± 12.0** | **0.844** |
| **Glinide** | **8.6 ± 1.5** | **7.7 ± 1.1** | **0.057** |  | **106.2 ± 13.5** | **105.9 ± 14.8** | **1.000** |
| **GLP1 RA** | **8.9 ± 1.7** | **7.8 ± 0.6** | **0.375** |  | **108.0 ± 29.9** | **108.4 ± 21.8** | **1.000** |
| **Sarcopenia** |  |  |  |  |  |  |  |
| **Insulin** | **8.9 ± 1.8** | **7.3 ± 0.8** | **0.500** |  | **101.1 ± 6.9** | **102.7 ± 8.2** | **0.750** |
| **Biguanide** | **10.5 ± 1.9** | **7.7 ± 0.4** | **0.062** |  | **99.0 ± 8.4** | **101.5 ± 8.4** | **0.438** |
| **DPP-4 inhibitor** | **10.0 ± 1.8** | **7.1 ± 0.7** | **0.002** |  | **99.9 ± 9.1** | **101.4 ± 5.6** | **0.679** |
| **Glinide** | **11.4 ± 0.5** | **7.8 ± 0.3** | **0.500** |  | **93.5 ± 11.3** | **99.9 ± 12.2** | **0.500** |
| **GLP1 RA** | **11.2 ± 1.3** | **7.9 ± 1.2** | **0.500** |  | **94.9 ± 33.0** | **98.2 ± 20.3** | **1.000** |

HbA1c, glycated hemoglobin; GNRI, Geriatric nutritional risk index; DPP4, dipeptidyl peptidase 4; SGLT2, sodium–glucose cotransporter 2; GI, glucosidase inhibitor; GLP-1RA, glucagon-like peptide-1 receptor agonists.

Supplementary Table 2. Changes in Albumin and BMI to the effect of additional glucose-lowering drugs treatment

|  | **Albumin (g/dL)** | | |  | **BMI (kg/m2)** | | |
| --- | --- | --- | --- | --- | --- | --- | --- |
|  | **baseline** | **12 months** | ***p*-value** |  | **baseline** | **12 months** | ***p-*value** |
| **Total** |  |  |  |  |  |  |  |
| **Normal** | **4.0 ± 0.4** | **4.1 ± 0.4** | **0.190** |  | **23.9 ± 4.4** | **23.8 ± 4.2** | **0.421** |
| **Sarcopenia** | **3.8 ± 0.5** | **4.0 ± 0.4** | **0.034** |  | **20.5 ± 3.1** | **21.1 ± 2.7** | **0.009** |
| **Male** |  |  |  |  |  |  |  |
| **Normal** | **4.1 ± 0.4** | **4.1 ± 0.4** | **0.290** |  | **23.3 ± 4.0** | **23.3 ± 3.7** | **0.926** |
| **Sarcopenia** | **3.8 ± 0.5** | **3.9 ± 0.4** | **0.039** |  | **20.1 ± 3.1** | **21.1 ± 2.6** | **0.011** |
| **Female** |  |  |  |  |  |  |  |
| **Normal** | **4.0 ± 0.4** | **4.1 ± 0.4** | **0.374** |  | **24.8 ± 4.8** | **24.6 ± 4.8** | **0.173** |
| **Sarcopenia** | **3.9 ± 0.5** | **4.0 ± 0.3** | **0.269** |  | **20.9 ± 3.0** | **21.2 ± 2.8** | **0.170** |

BMI, body mass index
